# Brain Activation in Response to Visceral Stimulation in Rats with Amygdala Implants of Corticosterone: An fMRI Study

**DOI:** 10.1371/journal.pone.0008573

**Published:** 2010-01-05

**Authors:** Anthony C. Johnson, Brent Myers, Jelena Lazovic, Rheal Towner, Beverley Greenwood-Van Meerveld

**Affiliations:** 1 VA Medical Center, Oklahoma City, Oklahoma, United States of America; 2 Oklahoma Center for Neuroscience, University of Oklahoma Health Sciences Center, Oklahoma City, Oklahoma, United States of America; 3 Biological Imaging Center, Division of Biology, California Institute of Technology, Pasadena, California, United States of America; 4 Advanced Magnetic Resonance Center, Oklahoma Medical Research Foundation, Oklahoma City, Oklahoma, United States of America; The Research Center of Neurobiology-Neurophysiology of Marseille, France

## Abstract

**Background:**

Although visceral pain of gastrointestinal (GI) origin is the major complaint in patients with irritable bowel syndrome (IBS) it remains poorly understood. Brain imaging studies suggest a defect in brain-gut communication in IBS with a greater activation of central arousal circuits including the amygdala. Previously, we found that stereotaxic implantation of corticosterone (CORT) onto the amygdala in rats induced anxiety and colonic hypersensitivity. In the present study we used functional magnetic resonance imaging (fMRI) to identify specific brain sites activated in a rat model characterized by anxiety and colonic hypersensitivity.

**Methodology/Principal Findings:**

Anesthetized male rats received micropellets (30 µg each) of either CORT or cholesterol (CHOL), to serve as a control, implanted stereotaxically on the dorsal margin of each amygdala. Seven days later, rats were anesthetized and placed in the fMRI magnet (7T). A series of isobaric colorectal balloon distensions (CRD - 90s ‘off’, 30s ‘on’, 8 replicates) at two pressures (40 and 60 mmHg) were performed in a standard block-design. Cross correlation statistical analysis was used to determine significant differences between distended and non-distended states in CORT and CHOL-treated animals. Analysis of the imaging data demonstrated greater overall brain activation in response to CRD in rats with CORT implants compared to CHOL controls. Additionally, CORT implants produced significant positive bilateral increases in MRI signal in response to CRD in specific nuclei known as integration sites important in anxiety and pain perception.

**Conclusions and Significance:**

These data indicate that chronic exposure of the amygdala to elevated levels of CORT enhances overall brain activation in response to CRD, and identified other specific brain regions activated in response to mechanical distension of the colon. These results demonstrate the feasibility of performing fMRI imaging in a rodent model that supports clinical observations in IBS patients with enhanced amygdala activation and symptomatology of abdominal pain and anxiety.

## Introduction

The role of the brain in the processing of information from the viscera, including the gastrointestinal (GI) tract, remains poorly understood. Clinical observations suggest that episodes of stress worsen symptoms associated with irritable bowel syndrome (IBS), a functional GI disorder characterized by episodes of abdominal pain, diarrhea and/or constipation and there is a close overlap between IBS and anxiety disorders [1]–[4]. Studies in patients with IBS suggest that heightened pain sensation to intraluminal distension of the colorectum may play a significant role in IBS symptomatology [5]–[7]. Altered perception of visceral stimuli due to afferent sensitization can occur due to abnormalities in ascending visceral signal processing from the gut to the brain [8], [9]. In addition, activation of central mechanism(s) resulting in colorectal hypersensitivity due to descending facilitation from the brain induces remodeling of colorectal responsiveness to distension and involves activation of supraspinal nuclei leading to sensitization of spinal dorsal horn neurons [10]–[13]. Clinically, activation of descending facilitatory pathways may play a role in the exacerbation of IBS symptoms in response to stress and anxiety [14], [15]. The amygdala, as part of the limbic system, is involved in the processing of visceral information, attention, emotion and integrating the physical and psychological components of the stress response. Additionally, the amygdala plays a crucial role in the generation and development of fear and anxiety [16], [17]. Specifically, the central nucleus of the amygdala (CeA) has been shown to facilitate the activation of the hypothalamic-pituitary-adrenal axis in response to stress and increase the release of corticotrophin releasing factor, adrenocorticotropic hormone, and corticosterone (CORT) [18], [19]. The CeA is also a major source of efferent pathways from the amygdala [20], and electrical stimulation of the CeA has been shown to modulate cardiovascular [21], respiratory [22], and GI function [5].

In recent years to investigate how the brain controls the GI tract, imaging studies using both functional magnetic resonance imaging (fMRI) and positron emission tomography have demonstrated that, in IBS patients, colorectal distension (CRD) activates areas of the brain involved in emotional sensory processing, particularly the amygdala, insula, and prefrontal cortex [23]–[25]. Although fMRI is a powerful tool for noninvasive imaging of brain function studies, neuroimaging in small animals such as rats are limited. However, brain imaging in rodent models offer many advantages such as being able to track the course of a diseases and examine the efficacy of novel treatments with control over relevant factors and experimental conditions that are uncontrollable in human studies. Previous studies to examine brain-gut communication and visceral pain pathways have used techniques ranging from electrophysiology [26]–[28] and c-fos detection [29]–[31] to behavioral analyses [32], [33]. One study using fMRI was able to detect brain areas activated by distension of the colorectal region in normal adult anesthetized rats [30]. The aim of our study was to use MRI signal to investigate the effect on supraspinal neuronal processing of manipulation of the amygdala via exposure to elevated levels of CORT shown previously to induce anxiety-like behaviors and increase the sensitivity of the colorectum to luminal distension after 7 days. Blood oxygenation level dependent (BOLD) fMRI in the brain was measured using T_2_^*^-weighted fast low angle shot (FLASH) imaging at 7T and was performed before, during and after CRD in rats with micropellet implants of CORT or cholesterol (CHOL) as a control. To our knowledge this is the first study to image specific brain regions in response to a defined manipulation of the amygdala in an animal model that mimics aspects of IBS and is characterized by heightened anxiety and abdominal pain. Our results showed that there are significant differences in brain activation in rats in response to modulation of the amygdala via elevated levels of CORT. These results complement recent studies from our group and support clinical observations in which abnormalities in amygdala activation appear common in IBS patients [24], [32], [34].

## Results

### Effect of CRD on Total CNS Activation in Rats with Elevated Levels of Amygdala CORT

**Figure 1:**
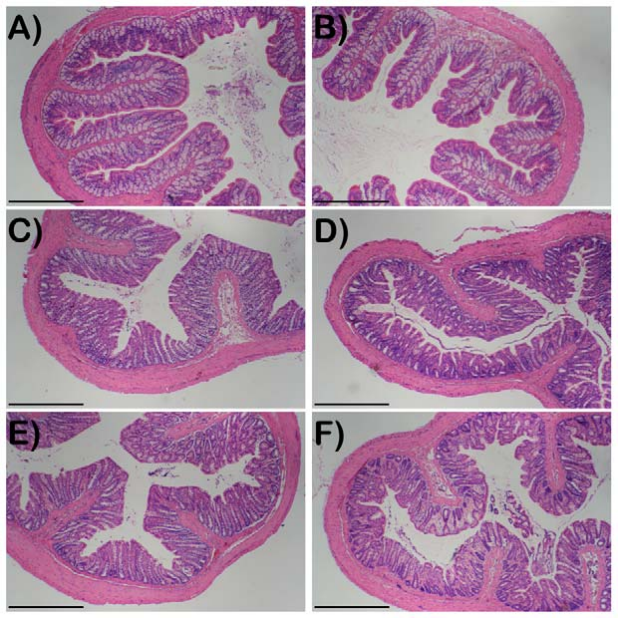
Histological appearance of the colonic mucosa from rats in which the balloon catheter was distended for 8 cycles ‘on’ and 8 cycles ‘off’ at 40 and 60 mmHg. A), C) and E) are from the proximal, medial and distal colon, respectively, of naïve rats. B), D) and F) are from the proximal, medial and distal colon, respectively, of rats that underwent the distention protocol. All slides are at 64× and the black bar represents 0.5 mm.

Initially we investigated whether repetitive CRD at 40 mmHg and 60 mmHg causes any disruption of the colonic mucosa. In this part of the study we used the same experimental design as that employed in the fMRI investigation, and examined the histological appearance of the colonic mucosa from rats that underwent CRD and compared the findings to naïve rats. Our results showed that 8 cycles of CRD at 40 mmHg and then 8 cycles at 60 mmHg caused no significant alteration in the architecture of the colonic mucosa compared to naïve controls (Figure 1) in any colonic region examined and confirmed that there was no change in the histological score between control and treated rats (Score = 0 for both groups in all regions).

**Figure 2:**
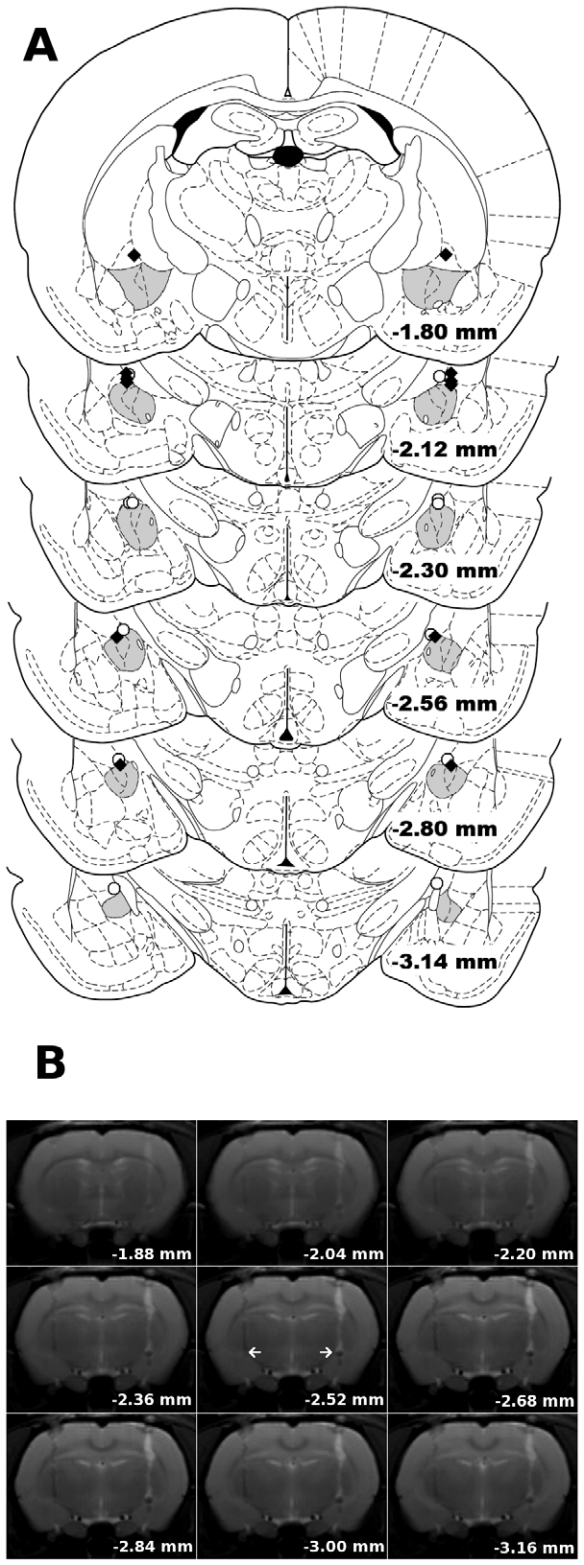
Localization of the micropellets. A) Adapted coronal diagrams from Paxinos and Watson [55] with the amygdala shaded. All micropellets (CHOL - white circles; CORT - black triangles) were on the dorsal margin of the amygdala between 1.80 and 3.14 mm caudal to bregma. B) Representative RARE images from a typical experiment showing the location of the micropellet over the dorsal margin of the amygdala. Distance is relative to bregma and the white arrows indicate the end of the cannula.

**Figure 3:**
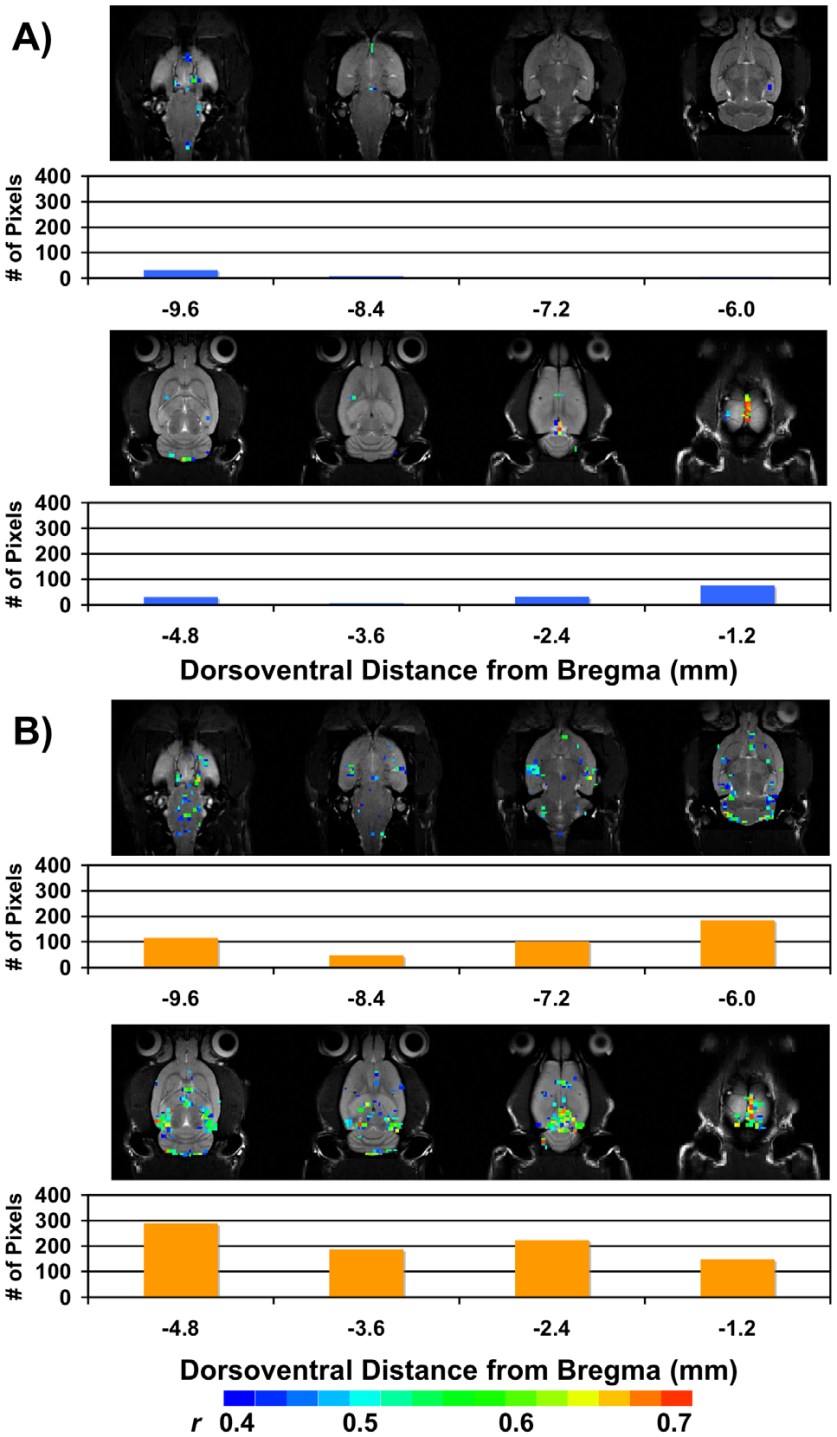
Total brain activation at 40 mmHg CRD. A) Activation in all rats with CHOL-containing micropellets. B) Activation in all rats with CORT-containing micropellets. For both A) and B), the *total* number of pixels per slice is shown in the histogram below the slice. The scale for *r* (0.4, blue to 0.7, red) is also provided. As illustrated, there was greater activation in rats with CORT micropellets in each slice.

**Figure 4:**
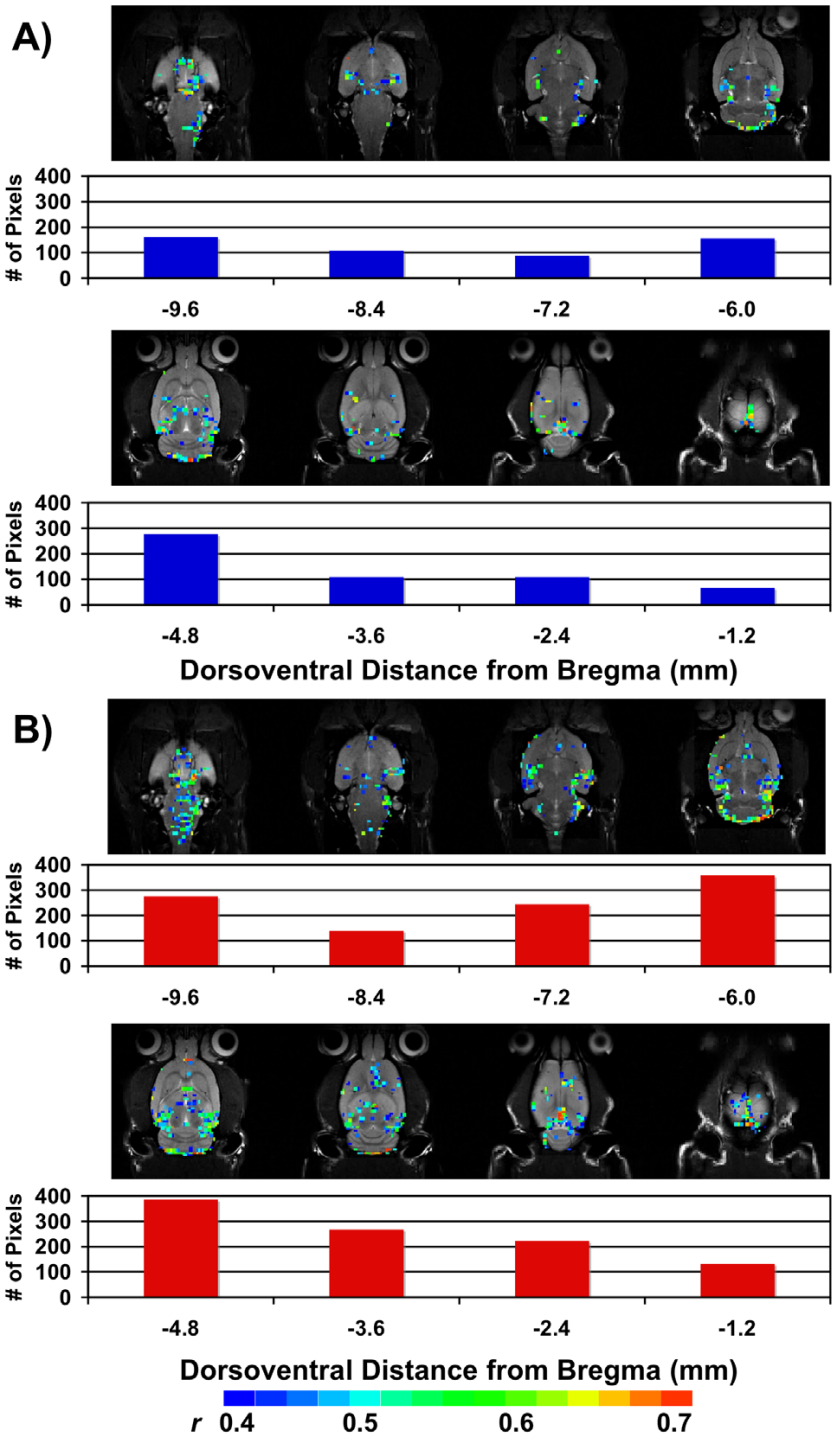
Total brain activation at 60 mmHg CRD. A) Activation in all rats with CHOL-containing micropellets. B) Activation in all rats with CORT-containing micropellets. For both A) and B), the *total* number of pixels per slice is shown in the histogram below the slice. The scale for *r* (0.4, blue to 0.7, red) is also provided. As illustrated, there was greater activation in rats with CORT micropellets in each slice.

**Figure 5:**
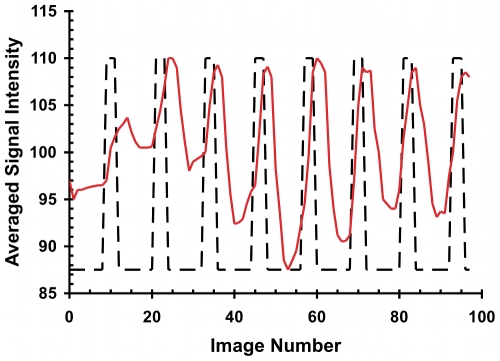
Change in signal intensity with CRD. The solid line represents the averaged fMRI signal intensity over the time-course of the distension paradigm (dashed-line represents deflation or inflation state of the colorectal balloon) for a typical experiment. As shown there is a rapid increase in signal intensity with balloon inflation indicating that the activated areas are due to the colonic stimulus.

In the current study we investigated total brain activation in response to 40 and 60 mmHg of CRD in anesthetized rats with bilateral implants of CORT placed stereotaxically on the dorsal margin of the amygdala (Figure 2). At 40 mmHg, CRD induced increases in CNS activation in control rats as illustrated in Figure 3A. The data also showed increases in the total number of activated pixels in CORT-implanted rats in response to CRD at 40 mmHg (Figure 3B). At a higher distension pressure of 60 mmHg there was an additional increase in the total number of activated pixels in CHOL controls (Figure 4A). The 60 mmHg CRD pressure also induced more total brain activation in rats with CORT implants (Figure 4B). A careful comparison of the average number of fMRI activated pixels between the 4 groups (CHOL, 40 mmHg – 28.2±14.3 pixels or 60 mmHg – 173.0±75.4 pixels vs. CORT, 40 mmHg – 201.8±82.3 or 60 mmHg – 317.0±115.5 pixels, n = 6 per group) indicated a significant effect of group (2-Way ANOVA, F = 4.442, *P*<0.05) and pressure (2-Way ANOVA, F = 4.548, *P*<0.05) on brain activation. However, post-hoc tests failed to demonstrate further significant interactions between the groups. In addition, the data demonstrated a similar maximum signal intensity change between the CORT and CHOL groups for both the 40 and 60 mmHg pressures of luminal distension. A typical time curve of fMRI signal intensity of all activated areas is shown in Figure 5.

### Specific Brain Nuclei Activated in Response to CRD in Rats with Elevated Levels of Amygdala CORT

**Figure 6:**
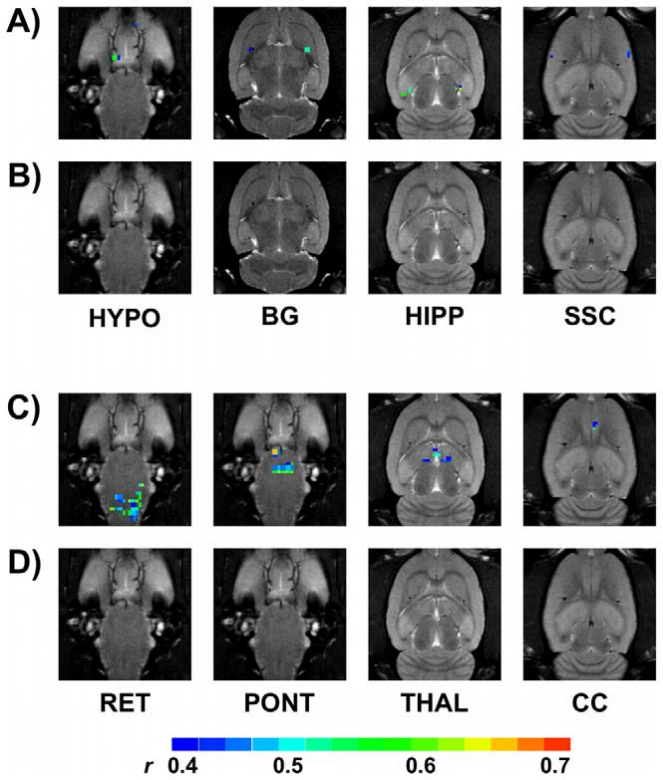
Specific nuclei activated in rats with CORT micropellets but not activated in rats with CHOL micropellets by 40 mmHg or 60 mmHg CRD. Illustrated data is from an individual rat representative of the median activation for either CORT (A and C) or CHOL (B and D). In the CORT treated rat, specific nuclei showing activation by CRD at 40 mmHg include the hypothalamus (HYPO), basal ganglia (BG), hippocampal complex (HIPP) and somatosensory cortex (SSC). In CORT-treated rat, specific nuclei showing activation by CRD at 60 mmHg include reticular nuclei (RET), pontine nuclei (PONT), thalamus (THAL) and the cingulate cortex (CC). The scale for *r* (0.4, blue to 0.7, red) is also provided.

In view of our initial observation of a greater increase in total brain activation in response to CRD in rats with elevated amygdala CORT for 7 days, we attempted to systematically examine specific brain nuclei that showed activation in CORT-treated rats but not in CHOL-implanted rats to gain a more complete picture of the central nuclei activated by CRD in the 2 groups. We focused our analysis to structures identified by fMRI as activated in response to 40 mmHg CRD in CORT-implanted rats but not activated in CHOL controls as these brain sites may be pivotal for the sensitization of visceral responses (Table 1 and 2). Of particular interest, we found that areas involved in the processing of emotion and pain showed activation at 40 mmHg in CORT-implanted animals but no activation in CHOL-implanted controls. In CORT implanted rats, the 40 mmHg visceral stimulus activated specific limbic structures including the hippocampal complex and the hypothalamus. Additionally, brain regions that form descending efferent pathways such as the basal ganglia exhibited activation in MRI signal in CORT but not CHOL-treated rats in response to 40 mmHg. CORT-treated rats also displayed activation in the sensory processing areas such as the somatosensory cortex during the 40 mmHg CRD that were not activated at the same distension pressure in CHOL controls (Figure 6A–B). As illustrated in figure 6C–D we show the nuclei that were activated at 60 mmHg CRD following amygdala CORT treatment but showed no activation in response the same stimuli in CHOL-treated rats. In response to 60 mmHg animals receiving CORT showed activated pixels in limbic areas such as the cingulate cortex and sensory integration areas including the thalamus. Additionally, brainstem structures involved in sensory-motor integration including the pontine and reticular nuclei also showed activation in response to 60 mmHg CRD in CORT-treated animals but not CHOL controls (Table 1 and 2).

**Table 1:** Nuclei activated in ventral axial slices 1–4 (Bregma −9.6–−6.0 mm).

Distension Pressure	40 mmHg		60 mmHg	
Nuclei	CHOL	CORT	CHOL	CORT
Amygdala	0.5±0.5	5.5±3.5	10.0±4.0	6.7±4.6
Basal Ganglia	NA	6.5±3.1	3.5±2.2	6.5±2.7
Cerebellum* †	NA	20.2±9.2	20.8±10.8	37.3±16.0
Cochlear Nuclei*	NA	0.5±0.3	NA	2.8±1.6
Ectorhinal Cortex*	NA	0.8±0.5	NA	5.0±2.7
Entorhinal Cortex	NA	3.0±1.4	3.0±1.8	4.8±4.1
Facial Nucleus	0.5±0.5	0.7±0.4	0.3±0.3	1.7±0.9
Hippocampus	1.0±1.0	5.3±2.8	13.7±8.8	21.7±10.1
Hypothalamus	2.5±1.6	2.7±1.1	10.3±5.9	11.2±5.1
Inferior Colliculus*	NA	0.3±0.3	0.2±0.2	1.3±0.5
Insular Cortex	NA	2.2±1.0	0.5±0.5	3.2±2.6
Internal Capsule	NA	0.2±0.2	0.2±0.2	1.7±1.1
Lateral Lemniscus	NA	0.3±0.3	1.2±1.2	2.7±2.1
Olfactory Nuclei	0.3±0.3	2.0±2.0	0.7±0.5	2.3±1.5
Perirhinal Cortex*	NA	4.2±2.0	0.7±0.7	5.7±2.6
Piriform Cortex	NA	0.8±0.8	1.7±1.7	2.7±2.5
Pontine Nuclei	0.3±0.3	3.0±1.8	3.3±3.0	9.2±4.7
Raphe Nuclei	0.7±0.7	0.3±0.3	2.2±1.4	3.5±1.6
Reticular Nuclei	0.5±0.5	6.5±3.2	6.3+4.1	14.2±7.1
Somatosensory Cortex	NA	0.3±0.3	NA	1.3±0.7
Tegmental Nuclei	0.8±0.8	1.7±1.1	1.2±0.8	6.0±2.5
Temporal Assoc. Cortex	NA	NA	NA	4.3±2.7
Thalamus	NA	0.5±0.5	1.7±1.3	2.7±1.8
Trigeminal Nuclei	1.0±0.7	3.8±1.9	3.7±2.4	5.7±2.9

Values listed are mean±SEM for pixels with *r*≥0.4, n = 6/group. NA = no activation.

* *P*<0.05 between groups.

† *P*<0.05, between pressures, 2-way ANOVA.

**Table 2:** Nuclei activated in dorsal axial slices 5–8 (Bregma −4.8–−1.2 mm).

Distension Pressure	40 mmHg		60 mmHg	
Nuclei	CHOL	CORT	CHOL	CORT
Basal Ganglia	1.2±1.2	0.8±0.5	3.5±2.9	2.7±1.6
Cerebellum	8.0±5.2	39.3±18.6	29.8±13.4	53.5±18.4
Cingulate Cortex	0.8±0.8	4.2±2.5	1.8±1.3	4.5±2.2
Corpus Callosum	NA	1.7±1.7	NA	3.2±2.4
Ectorhinal Cortex	NA	5.7±3.3	4.2±1.9	7.5±3.5
Entorhinal Cortex	NA	10.2±4.3	6.0±3.3	6.0±2.9
Habenular Nucleus	NA	2.0±1.3	1.0±1.0	1.0±0.7
Hippocampus	0.7±0.7	17.7±11.6	7.5±4.5	17.7±7.8
Inferior Colliculus	0.3±0.3	4.5±2.1	1.7±1.7	3.5±1.7
Insular Cortex	NA	NA	0.3±0.3	0.2±0.2
Internal Capsule	0.3±0.3	NA	0.7±0.4	NA
Motor Cortex*	0.5±0.5	1.8±0.7	0.2±0.2	2.2±0.7
Orbitofrontal Cortex	NA	NA	NA	2.3±1.7
Perirhinal Cortex	NA	8.5±3.4	7.3±3.9	7.8±3.0
Prelimbic Cortex	NA	1.2±1.2	NA	2.7±1.7
Pretectal Nucleus	NA	0.5±0.5	2.5±2.0	2.0±1.4
Retrosplenial Cortex	8.2±5.2	18.3±7.7	11.5±3.7	12.0±4.2
Septal Nucleus	NA	2.8±2.5	NA	1.5±1.1
Somatosensory Cortex*	NA	4.3±1.7	3.5±2.6	6.5±2.1
Superior Colliculus* †	NA	2.0±1.6	0.7±0.7	8.3±2.5
Temporal Assoc. Cortex	NA	2.0±1.4	1.8±1.2	4.5±1.9
Thalamus	NA	3.0±1.7	4.0±2.5	3.5±1.6

Values listed are mean±SEM for pixels with *r*≥0.4, n = 6/group. NA = no activation.

* *P*<0.05 between groups.

† *P*<0.05, between pressures, 2-way ANOVA.

**Figure 7:**
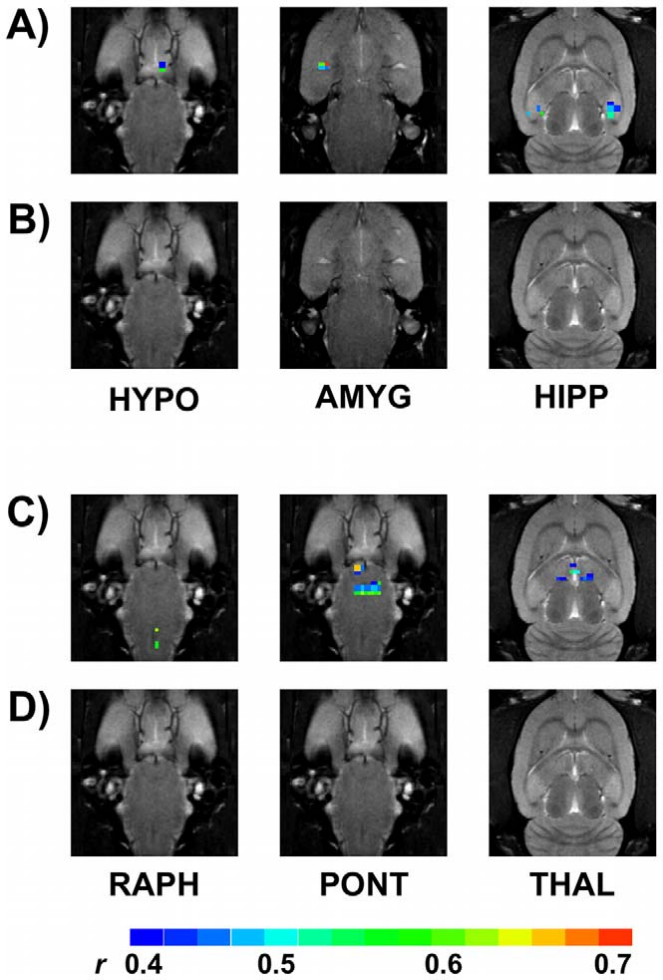
Specific nuclei activated by 60 mmHg CRD but not at 40 mmHg CRD in rats with either CORT or CHOL micropellets. Illustrated data is from an individual rat representative of the median activation. In the CHOL (A - 60 mmHg, B - 40 mmHg) treated rat, specific nuclei showing activation by CRD only at 60 mmHg include the hypothalamus (HYPO), the amygdala (AMYG) and hippocampal complex (HIPP). In the CORT (C – 60 mmHg, D – 40 mmHg) treated rat, specific nuclei showing activation by CRD only at 60 mmHg include the raphe (RAPH), pontine nuclei (PONT) and thalamus (THAL). The scale for *r* (0.4, blue to 0.7, red) is also provided.

Our final analysis examined specific central sites that were not activated in response to CRD at 40 mmHg but showed activation at the higher distension pressure of 60 mmHg in both CHOL and CORT treated rats (Table 1 and 2). This analysis demonstrated that the amygdala, hippocampus and hypothalamus, important regulators of autonomic and neuroendocrine function, showed activation only at 60 mmHg in CHOL-treated rats (Figure 7A–B). The pontine nuclei and thalamus showed activation at 60 mmHg but not 40 mmHg in CORT-implanted animals (Figure 7C–D). Interestingly, in CORT-treated rats, 60 mmHg distension also correlated with increased activation of the raphe nuclei which regulate serotonergic activity in the CNS.

## Discussion

Stress and anxiety alter visceral perception and may play a role in central pain amplification and the pathophysiology of IBS [15]. The current study used fMRI to identify areas of brain activation in response to distension of the colorectum. Specifically we investigated brain activation in response to CRD between rats with bilateral amygdala implants of CORT and age and weight-matched CHOL control rats. We provide evidence that elevated CORT on the amygdala significantly increases brain activation in response to CRD compared to that seen in CHOL-treated controls. Taken together the results of the present study support our hypothesis that exposure of the amygdala to elevated levels of CORT leads to colonic hypersensitivity via enhanced activation of specific central circuitry. Specifically, we showed that fMRI detects areas of the brain activated by CRD. In the current study we were able to show CNS activation in the CORT-treated animals in response to distension pressure (40 and 60 mm Hg) was significantly greater than that in CHOL controls. In light of the recent findings by Lazovic et. al. [30], also in anesthetized rats, where 40 mmHg distension pressures did not produce a significant activation of the brain, we opted not to attempt lower distensions pressures. The reason for the depressed threshold in the neuronal activation in response to CRD between the two studies is unknown but is unlikely to be related to the level of anesthesia since in both studies the rats were anesthetized with chloral hydrate. Another possible explanation may be related to the resolution of the images since we used a 7T magnet whereas the earlier study employed a 3T magnet. In the current study, the highest distension pressure that we employed was 60 mmHg, and we did not investigate CNS activation at higher distension pressures of 80 mmHg since there have been some reports that this level of CRD leads to sensitization of a visceromotor behavioral response, indicative of colorectal hypersensitivity that may be related to a local inflammatory response due to mucosal damage [35]. We found in the current study that repetitive CRD at 40 and 60 mmHg does not induce damage to the colonic mucosa thus eliminating the possibility that peripheral afferent sensitization was responsible for the enhanced brain activation.

A significant advance made in the current study was the enhanced brain activation in response to CRD in rats with CORT-containing micropellets placed stereotaxically on the dorsal margin of the amygdala. Thus, when taken together, we believe that this study provides pivotal data supporting the hypothesis that the amygdala is an important nucleus for the sensitization of neuronal sensory circuits that are involved in the induction of colonic hypersensitivity. The findings from the current study demonstrate a positive BOLD signal change in specific brain regions in response to elevated levels of CORT on the amygdala. Despite the size of the rat brain compared the human brain the use of a 7T magnet allowed us to distinguish specific brain nuclei but not sub-nuclei within that structure as seen with techniques such as c-fos and 2-deoxyglucose [36]. Here we show that CRD activated specific nuclei in both the CORT and CHOL controls. These areas include a collection of cortical and subcortical structures identified in human brain imaging studies as the pain matrix. This central network includes the thalamus, amygdala, cingulate cortex, basal ganglia, cerebellum and somatosensory cortex [37]–[39]. The current study demonstrates that, not only does luminal distension activate these structures in rodents, but also that CORT administration leads to a greater increase in the activation of these structures as well as the recruitment of other brain areas that were not activated in CHOL controls. A specific brain area activated in our study with particular relevance to visceral sensitivity is the hypothalamus which is known to be involved in emotional expression and the regulation of behavioral, neuroendocrine, and autonomic functions; however, this structure also contains visceral motor areas and plays an important role in modulating ascending nociceptive transmission. Specifically, the hypothalamus has diffuse connections to brainstem areas regulating visceral pain processing including the periaqueductal gray, raphe nuclei, and locus coeruleus [40]. Another structure important for visceral sensory processing is the cingulate cortex, which is involved in the awareness and perception of internal bodily states and contains specific subareas for integrating visceral sensation [41]. The cingulate also has direct connections to the amygdala, periaqueductal grey, and orbitofrontal cortex [42]. Taken together our observations in rodents [32], [34] support clinical observations in which abnormalities in amygdala activation appear common in IBS patients [24]. In the current study negative BOLD signal changes have not been analyzed since the interpretation of deactivation remains rather controversial, however they represent an area for future research investigation in the current animal model.

Although real-time fMRI has been reported in conscious rats by the team of Ferris and co-workers [43]–[45] who acclimated rats to the experimental conditions within the fMRI magnet as demonstrated by a reduction in the elevation in plasma CORT and motor movements, many technical and ethical challenges exist in performing experiments in unanesthetized rats using our experimental paradigm. To perform CRD in awake rats, they must be restrained in the fMRI magnet which is problematic in Fischer-344 (F344) rats for two important reasons: firstly there is a strong relationship between stress and the stimulation of visceral hypersensitivity in response to the stress provoking situations, and secondly F344 rats display a vulnerability to repeated stress since they do not habituate to the stressor whereas Sprague Dawley rats show a gradual decrease in the HPA axis following repeated restraint stress [46]. In light of these significant issues, we performed experiments in anesthetized animals, and given the robust nature of the BOLD signal intensity in our anesthetized animals in response to CRD we consider that studies on awake, restrained rats will likely yield data that is confounded by the effects of motion artifact during the distension procedure, stress due to the restraint and the inability to habituate F344 to the stressor. Recent studies claimed to have performed functional brain activation in unrestrained conscious rats in response to CRD [47], however the results from these studies must be treated with caution. Specifically, a limitation in the interpretation of these studies is that although [^14^C]-iodoantipyrine was injected after the onset of CRD in the conscious rats, the brain images were taken from autoradiographic analysis from the same animals following euthanization, which may have had an effect on the distribution of this marker. In the current study our experimental procedure of performing real-time imaging rather than post-mortem analysis of brain slices offers many significant and obvious advantages, however there are factors that may affect interpretation of our findings including potential changes in cerebral metabolism and blood flow due to the anesthetic that is required to avoid movement artifacts. Moreover, BOLD imaging is not a direct measure of neuronal activity; instead a measure of oxygen utilization, which we assume correlates with metabolic activity.

We consider our findings with 40 mmHg to have produced some pivotal data related to the central sites that may be involved in central sensitization despite the absence of data using lower distension pressures. However, in our model it is important to mention that central disinhibition of afferent signaling can also occur which would result in the same ‘hypersensitive’ response. Furthermore, a limitation of the current study is that although our study provides objective data for the existence of neuronal hypersensitivity, the structure-function relationship of these activated regions was beyond the scope of the current study. There are a number of important aspects of our model that support the validity of our conclusions. The first relates to the specificity of the observed responses to the amygdala. Although the micropellet was placed stereotaxically on the dorsal margin of the amygdala and ensures that the CORT bathes the central amygdala without physical damage to the structure, the possibility exists that other areas adjacent to the amygdala that express steroid receptors such as the hippocampus and caudate putamen, could be involved in our responses due to spread of the CORT from the micropellet. However, in our previous study we systematically performed a series of ‘off-site’ controls to examine the effect of elevated CORT in areas adjacent to the amygdala and found that the effects were specific to the central amygdala and not due to diffusion to adjacent brain regions [32]. Furthermore, to substantiate our claim that the steroid micropellet remained confined to the area of the implant, others have shown a diffusion distance of 750 µm for a 30 µg micropellet [19] and 900 µm for a 150 µg cannula of CORT [48]. Moreover, although there is a close relationship between the amygdala and the ventrocaudal caudate putamen, previous studies using autoradiography have shown limited binding of CORT [49], due to minimal expression of GR with no appreciable MR in the ventrocaudal caudate putamen [50]. Finally, although CRD can trigger contraction of the anal sphincter, and contraction of this sphincter also induces brain fMRI activity, in the current study external anal sphincter contraction was not measured but the level of fMRI activity was likely identical in both CORT and CHOL-treated rats in response to CRD.

In conclusion, the present study provides evidence of increased brain activation, measured as increased MRI signal, in response to CRD in rats, following exposure of the amygdala to elevated levels of CORT for 7 days. Our findings demonstrate that CRD lead to activation of the brain in specific regions known to be involved in somatosensory processing, and that exaggerated brain activation in CORT-implanted animals is suggestive of heightened sensitivity of the brain-gut axis in response to modulation of the amygdala. In light of data from IBS patients demonstrating increased activation in many of the same brain regions that we have observed in the current study, we speculate that our findings in a rodent model may imply that abnormal amygdala activity is a key component to enhanced brain activation involved in the development of anxiety and gut hypersensitivity in IBS.

## Materials and Methods

### Ethics Statement

All experiments were in compliance with the National Institute of Health Guide for Care and Use of Laboratory Animals and the International Association for the Study of Pain Research Guidelines. The Animal Care and Use Subcommittee at both the Oklahoma City Veterans Affairs Medical Center and the Oklahoma Medical Research Foundation approved all the experimental procedures.

### Animals

Experiments were performed on male F344 rats weighing 250–310 g and 13–15 wk of age purchased from Charles River Laboratories (Wilmington, MA) and housed under standard conditions with 12-h light/dark cycle with unrestricted access to chow and water. Rats remained in the animal facility for at least 7 days before the experimental procedure, and all experiments were performed at the same time each day. Experiments were performed in a total of 6 rats per experimental group (CORT 40 and 60 mmHg; CHOL 40 and 60 mmHg). A sample size of 6 in each group was determined to have 90% power to detect the expected difference between means (two group Satterthwaite t-test with a 0.05 two-sided significance level) [32].

### Stereotaxic Surgery

Animals had micropellets (30 µg - diameter of 0.3 mm with a height of 1.0 mm) of either CORT or CHOL placed bilaterally on the dorsal margin of the CeA under ketamine (100 mg/kg intraperitoneal (IP)) and xylazine (10 mg/kg IP) anesthesia as previously described [32], [34]. Animals were then allowed seven days for recovery, during which time their behavior was observed to ensure that they were not in distress.

### Localization of Stereotaxic Implant

During the imaging protocol, high-resolution anatomical images (RARE imaging sequence, field of view 2.4×2.4 cm^2^, matrix size 256×256) were obtained to illustrate the position of the micropellets of CORT or CHOL, as shown in Figure 2B. Following the imaging protocol, animals were euthanized and brains rapidly removed and frozen in chilled 2-methylbutane (Fisher Scientific, Fair Lawn, NJ). Brains were then stored at −80°C until cryosectioning. Serial coronal cross-sections (50 µm) were cryosectioned (Bright OTF, Fairfield, NJ) at −20°C and mounted onto slides followed by verification of micropellet placement by light microscopy.

### Colorectal Distension

A 5-cm latex balloon catheter was constructed as described previously [32]. The balloon was then inserted via the anal canal 8 cm into the colon and secured with surgical tape around the tail following choral hydrate anesthesia. CRD was performed by inflating the balloon using a constant pressure barostat (Model IIR, G&J Electronics, Toronto, Canada) synchronized to the fMRI magnet. A series of 8 phasic CRD was performed at distension pressures of 40 mmHg and then 8 phasic CRD at 60 mmHg for 30 s each (‘on’) with a 90 s deflation (0 mmHg – ‘off’) between distension periods.

### Histology

To determine whether CRD had any effect on the colonic mucosa, we performed the identical distension protocol in a subgroup of rats (n = 3). Naive rats (n = 3) in which a balloon was neither inserted nor distended served as controls. Three 1 cm tissue segments from each rat were collected from the proximal (1 cm distal to cecum), medial (8 cm proximal to rectum) and distal (2 cm proximal to rectum) colon. Tissues were fixed in 4% buffered formalin, processed, embedded in paraffin and sectioned at 5 µm thickness. They were stained with haematoxylin and eosin and assessed for inflammation by a blinded observer under a light microscope. Inflammatory parameters were scored using the following criteria: 0–2 for ulceration and fibrosis and 0–3 for inflammatory infiltration and depth of lesion [51]. A score of 0 designated no visible signs of pathology in the tissue.

### Functional Magnetic Resonance Imaging

Imaging was performed on a 7T Bruker MRI spectrometer (Biospec, Bruker Biospin, Billerica, MA) with a gradient coil (S116, Bruker Biospin). Prior to imaging, rats were anesthetized with chloral hydrate (400 mg/kg IP). To ensure a similar level of anesthesia across animals and within the same animal during the course of a single experiment an IP Teflon catheter (BD Insyte Autoguard, BD Medical Systems, Sandy, UT) was attached to an injection line for delivering additional anesthetic (40 mg/kg) if indicated by an increase in respiration rate by more than 20 breaths/min from the start of the experiment via monitoring respiration on a monitor (Model 1025, SA Instruments, Inc., Stony Brook, NY) via a transducer placed under the rat. The balloon catheter was then inserted and attached to the barostat with tygon tubing (4/16″ ID, 5/16″ OD). Following balloon catheter insertion, the rats were placed in a positioning frame and in the imaging coil (receive only quadrature surface coil and multirung volume coil for excitation). The barostat was interfaced to the MRI system console (MRI Interface, G & J Electronics, Inc.) to synchronize distention and deflation cycles with image acquisition. The fMRI paradigm consisted of 8 inflation and deflation cycles consisting of a 90 s baseline period with the balloon deflated (‘off’), during which time 9 images were acquired, followed by a 30 s activation or inflation period (‘on’), during which 3 images were obtained, for a total of 16 min and 96 images for each pressure (40 or 60 mmHg). The desired pressure of CRD was achieved by rapidly inflating (<5 s) the balloon controlled by the barostat.

### fMRI Image Selection

To minimize signal loss due to susceptibility artifacts at air-tissue interfaces (sinus cavities), the axial plane was chosen. Eight axial (1.2 mm thick) slices were positioned relative to bregma at the following dorsoventral distances: −9.6, −8.4, −7.2, −6.0, −4.8, −3.6, −2.4 and −1.2 mm. Anatomical T_2_-weighted images (effective TE/TR = 50.78/2500 ms, field of view 4×4 cm^2^, matrix size 128×128, 8 averages) were acquired in 5.3 min using the rapid acquisition with relaxation enhancement imagining sequence. Functional images were obtained using a T_2_^*^-weighted FLASH (effective TE/TR = 14/160 ms, 64×64 matrix, 1 average) 10.24 s for one image, with the same slice position, slice thickness, and field of view as the anatomical images.

### Data and Statistical Analysis

Post processing and analysis of fMRI data were performed using the CCHIPS software [52]. Image co-registration and motion corrections were achieved with a pyramid co-registration algorithm [53]. Areas of activation in different regions acquired during 8 ‘off’ cycles and 8 ‘on’ cycles was cross-correlated with an idealized wave representative of the stimulation paradigm [54]. A cross-correlation coefficient (*r*) value of the pixels higher than *r*≥0.4 is considered statistically significant (corresponding to *P*<0.0001), and is shown as different color-coded pixels representing activation on the image. Brain areas that contained activated pixels were identified based on a stereotaxic atlas [55], i.e. images were overlaid with the neuroanatomical image for nuclei identification. Total numbers of activated pixels for brain structures that extended over more than one slice were determined by adding activated pixels per each slice. Total brain activation in CORT and CHOL-treated animals was assessed at each pressure in terms of the total number of activated pixels per animal. Since a Bartlett's test for variance indicated a significant difference in variance between groups, to compare total brain activation between the CORT and CHOL groups in different brain nuclei, the total pixels per nuclei were logarithmically transformed (with no activation treated as 0). The transformed data was then subjected to a two-way analysis of variance (ANOVA) (group and pressure) followed by a Bonferroni post-hoc analysis (GraphPad Prism v. 4.0c for Mac, San Deigo, CA). The non-transformed data for individual nuclei were expressed as mean±standard error of the mean (SEM).

### Drugs and Chemicals

CHOL, CORT and choral hydrate were obtained from Sigma-Aldrich (St. Louis, MO). CHOL and CORT were placed in the brain as micropellets of the dry powder of the chemical. Choral hydrate was dissolved in sterile saline to a concentration of 100 mg/mL (wt/vol). Ketamine (100 mg/mL vial) was obtained from Phoenix Pharmaceutical (St. Joseph, MO) and administered in combination with xylazine (20 mg/mL vial) acquired from Hospira (Lake Forest, IL).
